# Preliminary insights into artificial intelligence guided dosing in hypertension and diabetes: challenges and lessons learnt in a pilot feasibility study

**DOI:** 10.1093/jamiaopen/ooaf153

**Published:** 2026-01-10

**Authors:** Jennifer Sumner, Mehul Motani, Jaminah Mohamed Ali, Si En Lee, Danliang Ho, Dylan Hong Tai Tan, Lieng Hsi Ling, Andre Teck Huat Tan, Gim Gee Teng, Santosh Kumar Seetharaman, Satya Pavan Kumar Gollamudi, Lin Siew Chong, Dean Ho, Amartya Mukhopadhyay

**Affiliations:** Medical Affairs – Research, Innovation & Enterprise, Alexandra Hospital, National University Health System, Singapore; Department of Electrical & Computer Engineering, National University of Singapore, Singapore; Institute of Data Science, Institute for Digital Medicine, N.1 Institute for Health, National University of Singapore, Singapore; Medical Affairs – Research, Innovation & Enterprise, Alexandra Hospital, National University Health System, Singapore; Department of Electrical & Computer Engineering, National University of Singapore, Singapore; Department of Electrical & Computer Engineering, National University of Singapore, Singapore; Department of Electrical & Computer Engineering, National University of Singapore, Singapore; Yong Loo Lin School of Medicine, National University of Singapore, Singapore; Department of Cardiology, National University Heart Centre, Singapore; Division of Endocrinology, Department of Medicine, National University Hospital, Singapore; Yong Loo Lin School of Medicine, National University of Singapore, Singapore; Chronic Programme, Alexandra Hospital, National University Health System, Singapore; Division of Rheumatology, Department of Medicine, National University Health System, Singapore; Healthy Ageing Programme, Alexandra Hospital, National University Health System, Singapore; Division of Geriatric Medicine, Department of Medicine, National University Hospital, Singapore; FAST Programme, Alexandra Hospital, National University Health System, Singapore; Division of Advanced Internal Medicine, Department of Medicine, National University Hospital, Singapore; Medical Affairs – Research, Innovation & Enterprise, Alexandra Hospital, National University Health System, Singapore; Institute of Data Science, Institute for Digital Medicine, N.1 Institute for Health, National University of Singapore, Singapore; Department of Biomedical Engineering, National University of Singapore, Singapore; Medical Affairs – Research, Innovation & Enterprise, Alexandra Hospital, National University Health System, Singapore; Yong Loo Lin School of Medicine, National University of Singapore, Singapore; Division of Respiratory and Critical Care Medicine, Department of Medicine, National University Hospital, Singapore

**Keywords:** Chronic disease management, ambulatory care, self-management, artificial intelligence, personalised medicine

## Abstract

**Objective:**

CURATE.AI is an artificial intelligence platform enabling personalised drug dosing. Aims:

1) Determine the feasibility of using CURATE.AI in the outpatient setting.

2) Compare the consistency of CURATE.AI recommendations derived from different data sources.

3) Assess the alignment of physician and CURATE.AI dosing recommendations.

**Materials and Methods:**

We conducted a single-arm feasibility study involving type II diabetics and hypertensives recruited from a hospital’s outpatient clinics. Outcomes included recruitment and study completion rates, adherence to study protocols, patient satisfaction, consistency of CURATE.AI recommendations across data sources, and alignment with physicians’ dosing decisions. We calibrated CURATE.AI for each individual using three distinct datapoints that linked drug dose to clinical response. After calibration, participants entered a four-month active phase, receiving monthly CURATE.AI dosing recommendations.

**Results:**

Eighteen participants were recruited, and thirteen completed the study. Only three progressed to the active study phase, primarily due to insufficient dose adjustments required during the calibration phase. Adherence to scheduled visits was 76% and adherence to home monitoring averaged 81%. Barriers to adherence included technical issues and work-related conflicts. Participants expressed high satisfaction with monitoring and care ≥88%. Actionable dosing recommendations were generated for two of the three participants, with varying alignment to physician decisions depending on the data source used.

**Discussion:**

Calibration challenges emerged when applying AI-guided dosing in a chronic disease population. Limited dose titration opportunities and cautious clinical practice restricted the data generation needed for effective model calibration.

**Conclusions:**

This pilot demonstrates the feasibility of deploying CURATE.AI into outpatient care but underscores the importance of aligning data requirements with patient and clinical characteristics. Future studies should target newly diagnosed patient groups with greater dosing variability to optimise calibration and assess clinical utility.

## Introduction

Precision medicine is an approach that tailors treatments based on a person’s genetics, environment, and lifestyle factors, to improve patient outcomes and enhance quality of life.^1–5^ However, optimising treatment plans based on individual characteristics is incredibly complex.^6^ As Artificial Intelligence (AI) and Machine Learning approaches improve, understanding complex information and deriving actionable strategies is becoming more feasible.^7^ In the realm of precision medicine, AI has facilitated advancements in various domains, including diagnostics, pharmacogenetics, and more recently medication optimisation.^8–13^

CURATE.AI, an AI-driven, actionable dosing optimisation platform, was initially developed to enhance the precision of chemotherapy dosing.^14–16^ Since its inception, it has demonstrated its efficacy in optimising single and combination therapies in cancer and immunosuppressed patients.^14–18^ The algorithm uses a patient’s treatment responses to various drug dosages over time. By leveraging each individual’s drug dose-response relationship data, CURATE.AI factors in the unique characteristics of an individual. This tailored approach ensures optimal dosing, leading to improved dosing efficacy, a reduced need for laboratory tests, and the minimisation of side effects—all leading to improved patient care.^14–18^

While CURATE.AI has succeeded in various inpatient settings,^14–18^ its application in chronic disease care is only just being explored.^19^^,^^20^ Using CURATE.AI in the outpatient setting comes with many unique challenges. Clinicians have less control over patients’ actions in terms of managing their treatment regimens outside the hospital. Furthermore, chronic disease is largely lifestyle-driven, which varies over time, making dosing recommendations challenging. Collecting data on clinical response also becomes more challenging, as data can only be gathered during scheduled clinic visits or through home monitoring, which relies on patient compliance. Moreover, the previous studies on CURATE.AI have primarily focused on intravenous medications offering accurate bioavailability and facilitating high-precision modulation. In contrast, outpatient treatments mainly involve enteral dosing, which may have unpredictable bioavailability and limited dosing increments for certain medications. Considering these factors, we conducted a feasibility study to evaluate the practical application of CURATE.AI in an outpatient setting for chronic disease management. Specifically, we tested the system on patients with hypertension and type II diabetes, the two most common chronic conditions treated in the outpatient setting. The study aimed to:

1. Determine the feasibility of using CURATE.AI in the outpatient setting.
2. Compare the consistency of CURATE.AI recommendations derived from different data sources.
3. Assess the alignment of physician and CURATE.AI dosing recommendations.

## Materials and methods

We evaluated the CURATE.AI system in a single-arm feasibility study between February 2022 to October 2023. This study was not designed to assess the clinical effectiveness of the system but rather to test its feasibility in an outpatient setting.^20^ A team of seven physicians were briefed on the study protocol and the CURATE.AI system. The CURATE.AI system was designed for research purposes and lacked a direct end-user interface. Therefore, physicians liaised with the research team to share data and receive dosing recommendations after participants were recruited into the study. The study is reported according to the CONSORT 2010 statement: extension to randomised pilot and feasibility trials.^21^

## CURATE.AI

CURATE.AI uses a quadratic equation to come up with an individualised CURATE.AI profile, upon which dosing recommendations are made:^20^


$$R\left(C,\;t\right)\;=\;F\left(S',\;C,\;t\right)-\;F\left(S',\;t\right)\;=\;x_{0}\;+\;\sum x_{i}c_{i}\;+\;\sum y_{ii}c_{i}^{2}\;+\;\sum z_{ij}c_{i}c_{j}$$

*R(* ***C***,***t****)* represents the overall treatment response; *F(****S’****,* ***C****,* ***t****)* represents a diseased patient under treatment; *F(****S’***,***t****)* represents the diseased patient; ***S’*** comprises the disease mechanisms; ***C*** represents the drug type and dose, ***t*** shows that every term in the series can vary with time and should be continually re-calibrated with clinical or point-of-care data, *x_i_* is the patient response coefficient to drug *i* at concentration *c_i_*, and *z_ij_* is the patient response coefficient to the interaction of drug *i* and drug *j* at their respective concentrations. The human body responds to drug inputs in a nonlinear fashion with respect to drug *i*. Therefore, *y_ii_* represents a second order response to the drug concentration, *c_i_*.^20^

To calibrate the system for each patient, we required a minimum of three different data points on drug dosage and the corresponding clinical response (ie, blood pressure or blood glucose levels). Once calibrated, CURATE.AI can start making dosing recommendations. All recommendations were calculated within the standard dosing ranges for specific drugs.

### Participants

The study aimed to recruit forty patients (n = 20 hypertensives and n = 20 type II diabetics) from the outpatient clinics at Alexandra Hospital, Singapore. Pilot or feasibility studies^22^ typically report sample sizes between 12 to 36 per arm.^23^^,^^24^ To evaluate the feasibility of CURATE.AI, we selected a sample of 20 per study arm. However, recruitment ended before targets were reached due to recruitment challenges. Potential participants were identified by the team of treating physicians during routine clinic visits. If eligible, a research team member was informed, and they approached the participant to discuss the project and obtain informed consent. The eligibility criteria were as follows:

### Inclusion criteria

- Adult patients ≥ 21years (the legal age of adulthood in Singapore) with clinically diagnosed type II diabetes or hypertension.
- Expected to be followed up at Alexandra Hospital in the next 6-months.

### Exclusion criteria

- Patients with cognitive impairment.
- Patients with active cancer undergoing chemotherapy.
- Patients on haemodialysis or peritoneal dialysis.
- Pregnant patients.
- Patients whose medications for diabetes and hypertension are changed simultaneously during their first clinic visit.
- Serious concomitant disorders that would compromise the safety of the patient or their ability to complete the study, at the discretion of the investigator.

### Study procedure

**Figure 1.**
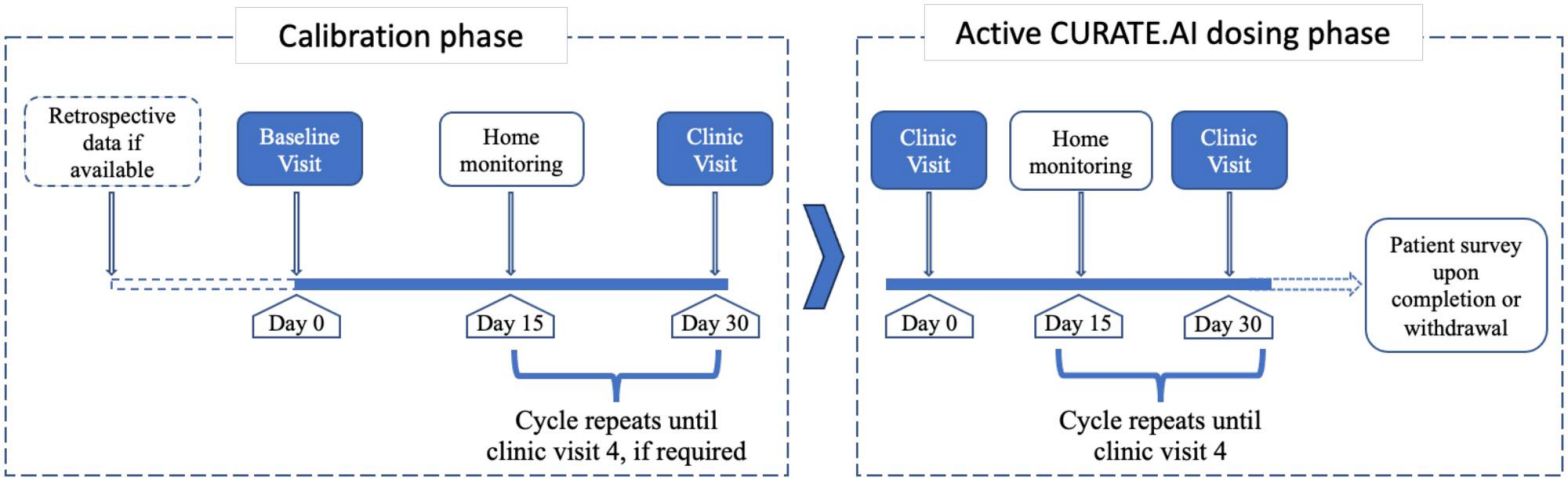
Study procedure overview.

The study flow is presented in Figure 1. At baseline, participants’ socio-demographics were captured by a research assistant (age, sex, marital status, employment status, educational status, and medical history) and clinical parameters were also measured, including height, weight, hip-waist ratio, blood pressure, renal function with estimated glomerular filtration rate, and liver function (aspartate aminotransferase and alanine aminotransferase). For the diabetic group only, glycated haemoglobin was also measured. During the monthly follow-ups, weight, glycated haemoglobin, blood pressure, changes in medication, renal and liver function were re-assessed. Home monitoring of blood pressure and glucose were conducted between clinic visits, and for those in the active phase of the study, disagreements between CURATE.AI recommendations and physician opinion were noted. At the end of the study or upon withdrawal from the study, each participant was invited to complete a patient satisfaction survey. The survey comprised of seven questions, scored on a 5-point Likert scale relating to their clinic visits and home monitoring experience. Survey completion was assisted by a research assistant.

#### Calibration phase (Figure 1)

Upon completion of baseline assessments, the participant enters the study at the calibration phase. To obtain the three dose-response calibration data points required by the platform, firstly, past medical records were checked for relevant dose-response data within the past 6-months. Second, up to four clinic visits were scheduled to obtain the necessary calibration data, if needed. In addition, home-monitoring of clinical parameters was conducted between the clinic visits. Hypertensive participants were asked to monitor their ambulatory blood pressure for one 6-hour period at home. A 6-hour ambulatory blood pressure recording has been found to be comparable to a 24-hour reading.^25^ However, due to poor compliance, there was insufficient data for analysis. Participants were then asked to continue blood pressure monitoring twice daily, before breakfast and dinner for up to seven days (Professional Blood Pressure Monitor, Omron, Kyoto, Japan). Diabetic patients were asked to wear a continuous glucose monitoring device (Continuous glucose monitoring system, Freestyle Libre, Abbott, Chicago, Illinois, United States) for at least seven days and up to fourteen days at home. Monitoring commenced approximately fifteen days after the clinic visit to allow any change in dose to be effective and stabilise. If calibration data could not be obtained within the calibration period, the participant exited the study at this point.

#### Active phase (Figure 1)

Upon calibration, participants entered the active phase, where CURATE.AI would generate dosing recommendations. Participants were assessed monthly (in-person or through tele-consultation) by the treating physician, with home monitoring in between (as per the calibration phase). At each clinic visit, a dosing recommendation was provided to the physician via the research team. As the clinical decision-maker, the physician determined the dose to prescribe to the participant, and the agreement or disagreement with CURATE.AI was recorded. Participants remained in the active phase of the study for a maximum of 4-clinic visits. If there was a change in another medication (not being optimised by CURATE.AI) related to the principal diagnosis under study, the participant was removed from the active phase as recalibration would be required. If a change of medication occurred that was unrelated to the principal diagnosis under study, then the patient remained in the active study phase.

### Data analysis

Feasibility was assessed using the following criteria: Whether recruitment targets were met (n = 40), the ability of participants to complete the calibration and active phases of the study, proportion of dropouts, compliance with clinic follow-ups (proportion of completed visits by number scheduled), compliance to home monitoring (proportion of completed home monitoring sessions by number scheduled), the proportion of disagreements between the CURATE.AI dosing recommendations and the physician’s opinion, and patient satisfaction. Data are presented as numbers and proportions as appropriate.

For CURATE.AI recommendations, the clinical data was utilised using four different approaches for hypertensive patients and three different approaches for diabetic patients (Table 1). This approach enabled us to compare the feasibility of different analytical approaches and the consistency in the CURATE.AI recommendations. Finally, as medications are only available in specific increments, the CURATE.AI recommendations were rounded to the nearest drug increment.

**Table 1.** Analysis approach used for clinical data.

Analysis approach	Definition
Hypertension	
Single systolic pressure readings	Data used from single clinic readings.
Single Mean Arterial Pressure (MAP) readings	MAP calculated from single clinic readings: MAP = Diastolic pressure + ⅓ (systolic pressure—diastolic pressure)
Systolic mixed data	Due to missing data, a mix of home monitoring and single clinic readings were combined. Home monitoring data was prioritised where feasible, using a median of the three most current data points.
MAP mixed	Due to missing data, a mix of home monitoring and single clinic readings were combined. Home monitoring data was prioritised were feasible, using a median of the three most current data points.
Diabetes	
HbA1c readings	Data used from clinic readings.
CGM data analysis	Using the three most recent days of CGM home monitoring data, the percentage of glucose readings that fell into the range of 4–10mmol/L were computed for each observation and then averaged.
Glycaemic variability data analysis	Using the three most recent days of CGM home monitoring data, glycaemic variability was computed for each observation and then averaged.

*Abbreviations*: MAP Mean Arterial Pressure; CGM Continuous glucose monitoring; HbA1c Glycated haemoglobin.

General clinical targets were defined as follows:

For diabetics:^26–28^

- HbA1c <7%
- Continuous glucose monitoring (CGM) -Time in range 4.0–10.0mmol/L >70% and/or Glycaemic variability ≤36%

For hypertensives:^29^^,^^30^

- Blood pressure in the clinic <130/80mmHg.
- Blood pressure from home monitoring (6-hr blood pressure or morning/afternoon measurement), average of readings < 135/85mmHg.
- Mean Atrial Pressure (MAP) <100mmHg.

The threshold for home monitoring was set higher due to account for the whitecoat effect.^31^ Tailored clinical thresholds were allowed for individual patients if specified by the treating physician.

## Results

We consented a total of eighteen participants, but one withdrew before the baseline assessments leaving seventeen participants. Table 2 shows the demographics of the seventeen who were enrolled and completed the baseline assessments.

**Table 2.** Participant demographics.

Demographics	*n* = 17
Sex: Male (%)	9 (53)
Age group, years (%):	
21–30	1 (6)
31–40	1 (6)
41–50	1 (6)
51–60	2 (12)
61 and older	12 (70)
Marital Status (%):	
Married	10 (59)
Single	7 (41)
Employment status (%):	
Employed	7 (41)
Unemployed or retired	10 (59)
Education Status (%):	
No formal/primary level education	8 (47)
Secondary	3 (18)
Diploma and above	6 (35)

### Recruitment and study completion

Eighteen participants consented to the study, reaching 45% of the original recruitment target. Challenges with eligibility were largely due to the stability of their presenting condition. After consent, one participant withdrew before baseline assessments, one participant passed away after two follow-up visits due to unrelated causes, and a further three withdrew after one follow-up, citing busy work schedules. This left thirteen participants who completed the study.

Of the eighteen recruited into the study, three completed calibration and reached the active phase of the study. Reasons for low conversion to the active phase were predominantly due to limited dose adjustment; hence, calibration data was not obtainable. Other issues included the requirement to restart the calibration phase if medications unrelated to the principal diagnosis changed, and the poor capture of clinical parameters necessary for calibration data.

### Adherence (n = 17)

Overall, adherence to scheduled clinic follow-ups was 76%. Most follow-up consultations were remote, creating unique challenges. For instance, some experienced technical issues with the teleconferencing software, requiring rescheduling of the appointment and delaying study procedures. Others missed their appointment if clinics did not run on time or if they could not find time during their work schedule to attend the follow-up (n = 3). Finally, some participants felt further appointments were unnecessary because of clinical stability (n = 3). Average compliance with home monitoring was 81%. Non-compliance with home-monitoring was due to the inconvenience of 6-hour ambulatory assessments, discomfort from the remote monitoring device, conflicts with personal plans, and the burden of repeated monitoring.

### Patient satisfaction (n = 17)

Although adherence to clinic visits and home monitoring was not always optimal, participants reported high satisfaction with both home monitoring and their care experience (Supplement 1). Most patients felt confident using the self-monitoring equipment (88% agreed), found it easy to integrate into daily life (100% agreed), and equipment issues were easy to resolve (100% agreed). Additionally, patients agreed that doctors provided clear treatment explanations (82% agreed), listened attentively (100% agreed), and trusted them with their care (100% agreed).

### CURATE.AI dosing decisions

**Figure 2.**
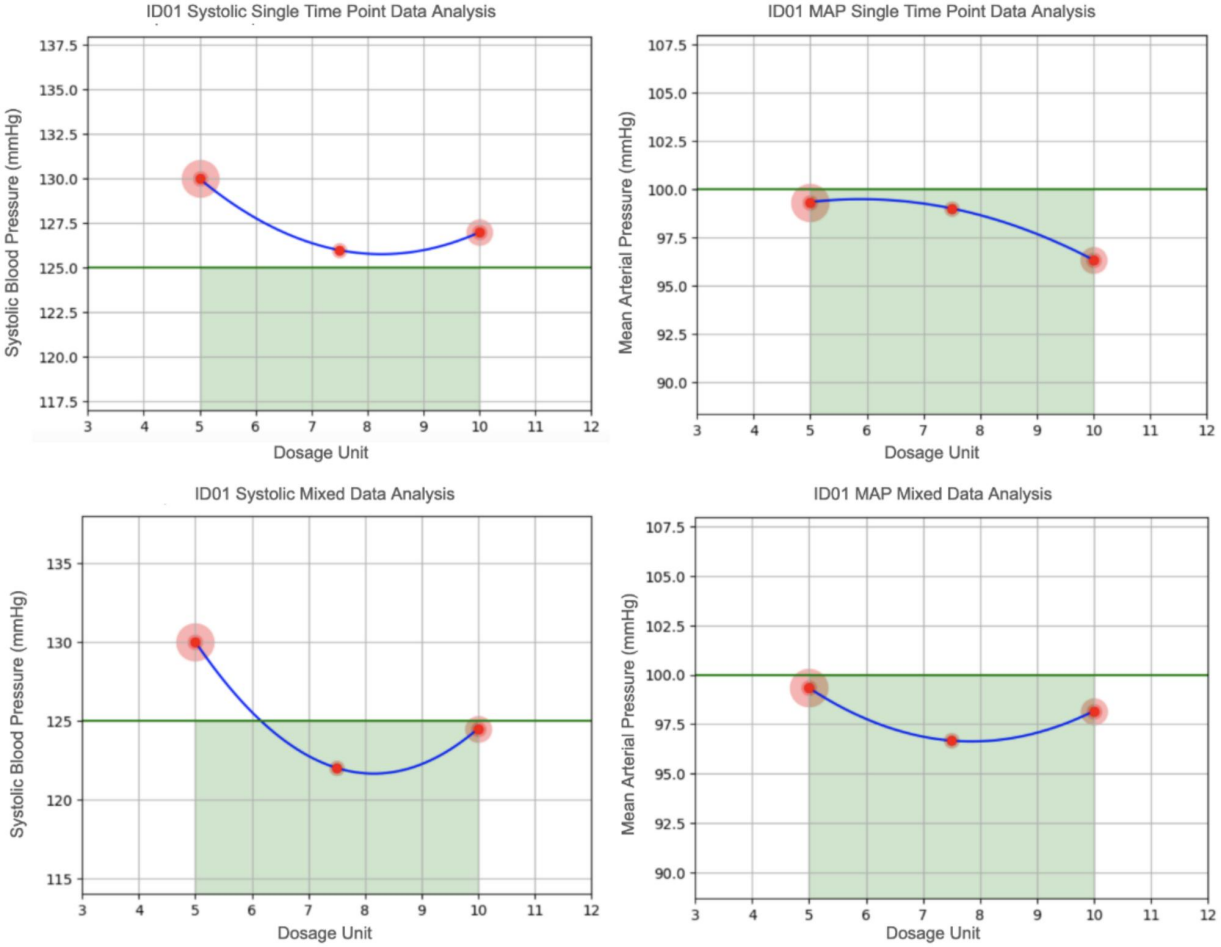
Plots of the CURATE.AI quadratic curve used to determine dosing recommendations for Amlodipine based on four different data sources, participant ID01 (Green line represents target clinical threshold aiming to be below, the lowest point in the curve represents the dosing recommendation). The blue curves in each plot represent the CURATE.AI plot, the green horizontal lines represent our target clinical metric, and the width of the green shaded area represents the acceptable dosing range (set a-priori based on standard dosing guidelines for each drug). The dose recommendation is derived from the lowest point on the curve that falls below the target clinical metric line (below 125mmHg for systolic blood pressure or 100mmHg for MAP, depending on the analyses).

**Figure 3.**
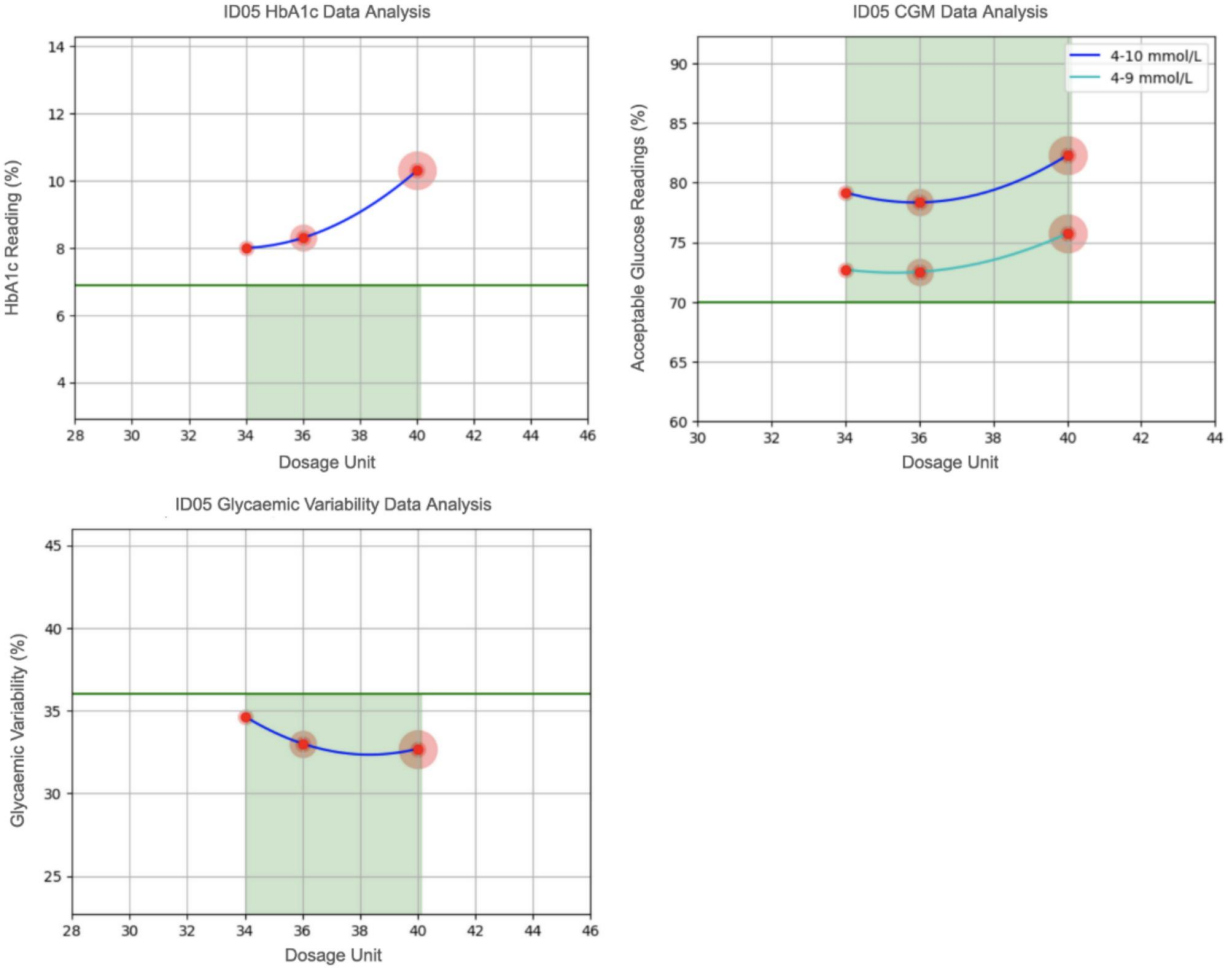
Plots of the CURATE.AI quadratic curve used to determine dosing recommendations for Novomix based on three different data sources, participant ID05 (Green line represents target clinical threshold aiming to be below, the lowest point in the curve represents the dosing recommendation).

Three patients (ID01 Male, 30-year-old hypertensive, ID02 Male, 56-year-old diabetic, and ID05 Female, 62-year-old diabetic) reached the active phase of the study and received CURATE.AI dosing recommendations. For each participant, we present a comparison of the CURATE.AI recommendations derived from the different analytical approaches (as outlined in Table 2), and the physician’s dosing recommendation (Tables 3 and 4 and Figures 2 and 3).

**Table 3.** Dosing recommendations for Amlodipine by CURATE.AI and physician decision at the four follow-up visits, participant ID01

Reading	Systolic single time point data analysis	MAP single time point data analysis	Systolic mixed data analysis	MAP mixed data analysis	Final CURATE.AI dosing decision	Physician dosing decision
1	Insufficient data	Insufficient data	7.5 mg	7.5 mg	Amlodipine 7.5mg	Amlodipine 7.5mg
2	Undefined	10 mg	7.5 mg	7.5 mg	Amlodipine 7.5mg	Amlodipine 7.5mg
3	7.5 mg	10 mg	7.5 mg	7.5 mg	Amlodipine 7.5mg	Amlodipine 7.5mg
4	7.5 mg	10 mg	7.5 mg	7.5 mg	Amlodipine 7.5mg	Amlodipine 7.5mg

Dosing recommendations based on four different forms of clinical data: (1) Single clinic readings, (2) MAP based on single clinic readings, (3) A mix of home monitoring and single clinic readings, (4) MAP based on a mix of home monitoring and single clinic readings.

MAP: Mean Arterial Pressure.

**Table 4.** Dosing recommendations for Novomix by CURATE.AI and physician decision at the four follow-up visits, participant ID05

Readings	HbA1c data analysis	CGM data analysis	Glycaemic variability data analysis	Curate.AI dosing recommendation	Physician dosing decision
**1**	Undefined	36 units	38 units	Novomix 36 – 38 units	Novomix 34 units
**2**	Undefined	36 units	38 units	Novomix 36 – 38 units	Novomix 34 units
**3**	Undefined	36 units	38 units	Novomix 36 – 38 units	Novomix 34 units
**4**	Undefined	36 units	38 units	Novomix 36 – 38 units	Novomix 38 units

Dosing recommendations based on three different forms of clinical data: (1) Data from clinic HbA1c readings, (2) Percentage of CGM glucose readings within the range 4–10mmol/L, (3) Average CGM glycaemic variability.

CGM: Continuous Glucose Monitoring.

For participant ID01, the treating physician set a specific clinical threshold for systolic blood pressure at 125 mmHg. In this case, the physician’s dosing decisions matched CURATE.AI recommendations at all four follow-up visits (Table 3).

For participant ID02, missing data restricted the analysis to HbA1c only, but no actionable CURATE.AI recommendations could be derived (Supplement 2). For participant ID05, CURATE.AI recommendations based on glucose data differed from the physician’s decision in 3 out of 4 follow-up visits, with CURATE.AI suggesting a higher dose than the physician preferred dose (Table 4). Like ID02, no actionable CURATE.AI recommendations were derivable from HbA1c readings. As shown in Figure 3, while recommendations could be derived from glucose data, the HbA1c quadratic curve did not fall below the clinical target (horizontal green line); thus, no recommendation was derivable.

## Discussion

The principal objective of this study was to assess the feasibility of using CURATE.AI in the outpatient setting to support chronic disease dosing. Our feasibility outcomes included recruitment, study completion, adherence and patient satisfaction indicators. Since most patients had stable blood pressure and blood sugar control, the main challenge of using CURATE.AI was obtaining enough calibration data. Logistically, the study procedure was feasible in terms of adherence to more frequent clinic visits and home-monitoring. Patients also aligned with this, reflecting positive care and home-monitoring experiences. Finally, we generated actionable recommendations from the CURATE.AI algorithm for two participants, demonstrating the feasibility of using CURATE.AI in the outpatient setting. Future work is needed to optimise workflows and ascertain what type and format of clinical data is most suitable for CURATE.AI.

The increasing strain on healthcare systems due to workforce shortages, short outpatient consult times, and a growing number of patients with chronic conditions necessitates innovative solutions to optimise care delivery.^32–35^ AI-guided dosing tools, such as CURATE.AI, can help improve efficiency by automating routine dose adjustments, allowing clinicians to focus on complex, high-need cases.^13–18^ Furthermore, AI can enable greater treatment precision by factoring in more of the unique factors that make up an individual and influence drug response.^14–18^ In doing so, patient outcomes can improve, and complications can be minimised, contributing to further workload reductions.

In this study, we demonstrated the potential of CURATE.AI in the outpatient setting. Overall, adherence to the study protocol was generally high. As monthly follow-ups did not require in-depth clinical assessments, appointments were succinct, minimising the burden on the treating physician. Additionally, by using virtual follow-up visits, study participation was made more convenient for patients. In time, as confidence in CURATE.AI grows, follow-up visits could be handled by other clinical staff, as is common practice for routine titration tasks.^36^ Anecdotally, we also found home-monitoring was not burdensome to physicians, who valued the additional data access that gave them greater insights for clinical decision-making and motivated compliance.

Despite good adherence to the protocol, we also faced several challenges. For instance, the inability to meet recruitment targets due to a lack of unstable patients requiring frequent dose modifications and the impact of COVID-19 on outpatient clinic visitation. Where suitable candidates were available, clinicians appeared hesitant to modify established dosing regimens more frequently than usual, likely reflecting an unconscious tendency to minimise clinical risk. This observation suggests that while physicians may, in principle, be willing to try new technology, engrained clinical practices take time to change.^37^ Finally, the range of tablet doses available is limited; this also likely contributed to infrequent dose changes. Deploying technologies like 3D drug printing could help overcome such limitations in the future by offering more diverse and precise tablet dosing options.^38^

The calibration challenges observed in this study point to the importance of aligning AI dosing tools with clinical context. AI dosing tools may be better suited to patients whose treatment plans involve ongoing dose titration, such as those newly diagnosed, where dosing adjustments occur more frequently and sufficient data can be gathered within a shorter period. These findings underscore the importance of aligning both study design and patient selection with the evolving data requirements of AI-based dosing systems.

Another area that requires careful consideration with AI tools is the data; the data source, and how it is analysed. In this study, we explored using data captured at the clinic and home monitoring data, as well as different ways of analysing this data for input into CURATE.AI. Analyses revealed variations in recommendations depending on the data input, creating uncertainty around what data source to use. We took a pragmatic approach and based final decisions on the largest consensus, but further work is needed to optimise the approach and identify which metric is most reliable. Another challenge was when patients had split dosing regimens (AM/PM). To generate a recommendation using CURATE.AI would require separate calibration data for AM and PM doses. In practice, this was infeasible, so daily AM and PM dosing data were summed up to generate a single dose. However, this subsequently required manually splitting the CURATE.AI recommendation back into an AM/PM arrangement. Finally, changes to other concurrent medications complicated calibration, as following other medication changes, further data was required to calibrate. This delay led to some patients never reaching the active phase of the study. While it is possible to calibrate multiple drugs concurrently with CURATE.AI we did not assess this in this feasibility study. Future work is needed to evaluate the practicalities of multiple concurrent drug optimisations in the context of outpatient care.

### Strengths and limitations

The main limitation of this study is the small sample size, a larger study is needed to draw more definitive conclusions. However, valuable lessons, particularly on the logistical aspects of the project, were still garnered and will be used to inform future work. Another limitation is that the physicians had no direct interaction with the CURATE.AI tool. This decision was made as the interface was not sufficiently developed to enable deployment. Thus, our feasibility study lacks data on the direct experiences of interacting with the CURATE.AI system. Finally, only three patients reached the active phase of the study. The findings from three participants, while positive, may not be representative.

### Conclusions

This pilot study demonstrates the feasibility of implementing CURATE.AI in the outpatient chronic disease management setting. The success of AI-driven dosing systems depends on careful patient selection, home monitoring capacity, and the availability of flexible dosing regimens. These early insights provide an overview of the practical challenges of integrating personalised dosing algorithms into clinical workflows and identify areas for further refinement.

## Supplementary Material

ooaf153_Supplementary_Data

## Data Availability

Data available on request.

## Code availability

Not applicable to this article.

## References

[1] König IR , Fuchs O, Hansen G, et al What is precision medicine? Eur Respir J. 2017;50:391. 10.1183/13993003.00391-2017 29051268

[2] McGrath S , Ghersi D. Building towards precision medicine: empowering medical professionals for the next revolution. BMC Med Genomics. 2016;9:23. 27160306 10.1186/s12920-016-0183-8 PMC4862053

[3] Chaikijurajai T , Laffin LJ, Tang WHW. Artificial intelligence and hypertension: recent advances and future outlook. Am J Hypertens. 2020;33:967–974. 32615586 10.1093/ajh/hpaa102 PMC7608522

[4] Bamba H , Singh G, John J, et al Precision medicine approaches in cardiology and personalized therapies for improved patient outcomes: a systematic review. Curr Probl Cardiol. 2024;49:102470. 38369209 10.1016/j.cpcardiol.2024.102470

[5] Sharma R , Musyuni P, Majeed J, et al Challenges and opportunities in precision therapy for diabetic patients. Health Sciences Review. 2024;12:100190.

[6] Lajmi N , Alves-Vasconcelos S, Tsiachristas A, et al Challenges and solutions to system-wide use of precision oncology as the standard of care paradigm. Cambridge Prisms: Precision Medicine. 2024;2:e4. 38699518 10.1017/pcm.2024.1 PMC11062796

[7] van Assen M , Lee SJ, De Cecco CN. Artificial intelligence from a to Z: from neural network to legal framework. Eur J Radiol. 2020;129:109083. 32526670 10.1016/j.ejrad.2020.109083

[8] Subramanian M , Wojtusciszyn A, Favre L, et al Precision medicine in the era of artificial intelligence: implications in chronic disease management. J Transl Med. 2020;18:472. 33298113 10.1186/s12967-020-02658-5 PMC7725219

[9] Bhinder B , Gilvary C, Madhukar NS, et al Artificial intelligence in cancer research and precision medicine. Cancer Discov. 2021;11:900–915. 33811123 10.1158/2159-8290.CD-21-0090 PMC8034385

[10] Beam AL , Drazen JM, Kohane IS, et al Artificial intelligence in medicine. N Engl J Med. 2023;388:1220–1221. 36988598 10.1056/NEJMe2206291

[11] Rajpurkar P , Chen E, Banerjee O, et al AI in health and medicine. Nat Med. 2022;28:31–38. 35058619 10.1038/s41591-021-01614-0

[12] Naik K , Goyal RK, Foschini L, et al Current status and future directions: the application of artificial intelligence/machine learning for precision medicine. Clin Pharmacol Ther. 2024;115:673–686. 38103204 10.1002/cpt.3152

[13] Ribba B , Dudal S, Lavé T, et al Model-informed artificial intelligence: reinforcement learning for precision dosing. Clin Pharmacol Ther. 2020;107:853-857. 31955414 10.1002/cpt.1777

[14] Blasiak A , Khong J, Kee T. CURATE.AI: Optimizing personalized medicine with artificial intelligence. SLAS Technol. 2020;25:95–105. 31771394 10.1177/2472630319890316

[15] Pantuck AJ , Lee D-K, Kee T, et al Modulating BET bromodomain inhibitor ZEN-3694 and enzalutamide combination dosing in a metastatic prostate cancer patient using CURATE.AI, an artificial intelligence platform. Adv Ther. 2018;1:1800104.

[16] Blasiak A , Tan LWJ, Chong LM, et al Personalized dose selection for the first waldenström macroglobulinemia patient on the PRECISE CURATE.AI trial. NPJ Digit Med. 2024;7:223. 39191913 10.1038/s41746-024-01195-5 PMC11350179

[17] Zarrinpar A , Lee DK, Silva A, et al Individualizing liver transplant immunosuppression using a phenotypic personalized medicine platform. Sci Transl Med. 2016;8:333ra49. 10.1126/scitranslmed.aac5954 27053773

[18] Tan SB , Kumar KS, Truong ATL, et al Comparing the performance of multiple small-data personalized tacrolimus dosing models for pediatric liver transplant: a retrospective study. Annu Int Conf IEEE Eng Med Biol Soc. 2023;2023:1–4. 10.1109/EMBC40787.2023.10341002 38083591

[19] Truong ATL , Tan SB, Wang GZ, et al CURATE.AI-assisted dose titration for anti-hypertensive personalized therapy: study protocol for a multi-arm, randomized, pilot feasibility trial using CURATE.AI (CURATE.AI ADAPT trial). Eur Heart J Digit Health. 2024;5:41–49. 38264697 10.1093/ehjdh/ztad063 PMC10802822

[20] Mukhopadhyay A , Sumner J, Ling LH, et al Personalised dosing using the CURATE.AI algorithm: protocol for a feasibility study in patients with hypertension and type II diabetes mellitus. Int J Environ Res Public Health. 2022;19:8979. 35897349 10.3390/ijerph19158979 PMC9332044

[21] Eldridge SM , Chan CL, Campbell MJ, et al CONSORT 2010 statement: extension to randomised pilot and feasibility trials. Bmj. 2016;355:i5239. 27777223 10.1136/bmj.i5239 PMC5076380

[22] Kunselman AR. A brief overview of pilot studies and their sample size justification. Fertil Steril. 2024;121:899–901. 38331310 10.1016/j.fertnstert.2024.01.040 PMC11128343

[23] Julious SA. Sample size of 12 per group rule of thumb for a pilot study. Pharm Stat. 2005;4:287–291.

[24] Billingham SAM , Whitehead AL, Julious SA. An audit of sample sizes for pilot and feasibility trials being undertaken in the United Kingdom registered in the United Kingdom clinical research network database. BMC Med Res Methodol. 2013;13:104. 23961782 10.1186/1471-2288-13-104 PMC3765378

[25] Ernst ME , Sezate GS, Lin W, et al Indication-specific 6-h systolic blood pressure thresholds can approximate 24-h determination of blood pressure control. J Hum Hypertens. 2011;25:250–255. 20574446 10.1038/jhh.2010.66 PMC2946963

[26] Battelino T , Danne T, Bergenstal RM, et al Clinical targets for continuous glucose monitoring data interpretation: recommendations from the international consensus on time in range. Diabetes Care. 2019;42:1593–1603. 31177185 10.2337/dci19-0028 PMC6973648

[27] Monnier L , Colette C, Wojtusciszyn A, et al Toward defining the threshold between low and high glucose variability in diabetes. Diabetes Care. 2017;40:832–838. 28039172 10.2337/dc16-1769

[28] Ministry of Health. Diabetes Mellitus MOH Clinical Practice Guidelines. Singapore: MOH; 2014. https://www.moh.gov.sg/docs/librariesprovider4/guidelines/cpg_diabetes-mellitus-summary-card—jul-2014.pdf? sfvrsn=be4a56e4_0.

[29] Ministry of Health. Hypertension MOH Clinical Practice Guidelines. Singapore: MOH; 2023. https://www.moh.gov.sg/hpp/doctors/guidelines/GuidelineDetails/cpgmed_hypertension.

[30] DeMers D , WD, Physiology. Mean Arterial Pressure. USA: Treasure Island (FL): StatPearls Publishing; 2023. 30855814

[31] Pickering TG , Gerin W, Schwartz AR. What is the white-coat effect and how should it be measured? Blood Press Monit. 2002;7:293–300. 12488648 10.1097/00126097-200212000-00001

[32] World Health Organization. Noncommunicable Diseases. Geneva: WHO; 2021. https://www.who.int/health-topics/noncommunicable-diseases#tab=tab_1.

[33] World Health Organization. Ageing and Health. Geneva: WHO; 2024. https://www.who.int/news-room/fact-sheets/detail/ageing-and-health.

[34] Azzopardi-Muscat N , Zapata T, Kluge H. Moving from health workforce crisis to health workforce success: the time to act is now. The Lancet Regional Health—Europe. 2023;35:100765. 38115956 10.1016/j.lanepe.2023.100765 PMC10730309

[35] Secinaro S , Calandra D, Secinaro A, et al The role of artificial intelligence in healthcare: a structured literature review. BMC Med. Inform. Decis. Mak. 2021;21:125. 33836752 10.1186/s12911-021-01488-9 PMC8035061

[36] Leong SL , Teoh SL, Fun WH, et al Task shifting in primary care to tackle healthcare worker shortages: an umbrella review. Eur. J. Gen. Pract. 2021;27:198-210. 34334095 10.1080/13814788.2021.1954616 PMC8330741

[37] Ubel PA , Asch DA. Creating value in health by understanding and overcoming resistance to de-innovation. Health Aff. 2015;34:239-244. 10.1377/hlthaff.2014.0983 25646103

[38] Alzoubi L , Aljabali AAA, Tambuwala MM. Empowering precision medicine: the impact of 3D printing on personalized therapeutic. AAPS PharmSciTech. 2023;24:228. 37964180 10.1208/s12249-023-02682-w

