# Supplementary material for: Preliminary insights into artificial intelligence guided dosing in hypertension and diabetes: challenges and lessons learnt in a pilot feasibility study
Source: JAMIA Open. 2026 Jan 10;9(1):ooaf153. doi: 10.1093/jamiaopen/ooaf153 (PMC12794016; doi:10.1093/jamiaopen/ooaf153)
Supplement: ooaf153_Supplementary_Data [file ooaf153_supplementary_data.zip › Supplement 2.pdf]

Dosing recommendations by CURATE.AI and physician decision at the four follow-up visits, participant ID02

Readings	HbA1c data analysis	CGM data analysis	Glycaemic variability data analysis	Curate.AI dosing recommendation	Physician dosing decision
**1**	Undefined	Insufficient data	Insufficient data	Undefined	Glargine 22U
**2**	Undefined	Insufficient data	Insufficient data	Undefined	Glargine 22U
**3**	Undefined	Insufficient data	Insufficient data	Undefined	Glargine 22U
**4**	Undefined	Insufficient data	Insufficient data	Undefined	Glargine 22U
