# Supplementary material for: Preliminary insights into artificial intelligence guided dosing in hypertension and diabetes: challenges and lessons learnt in a pilot feasibility study
Source: JAMIA Open. 2026 Jan 10;9(1):ooaf153. doi: 10.1093/jamiaopen/ooaf153 (PMC12794016; doi:10.1093/jamiaopen/ooaf153)
Supplement: ooaf153_Supplementary_Data [file ooaf153_supplementary_data.zip › Supplement 1.pdf]

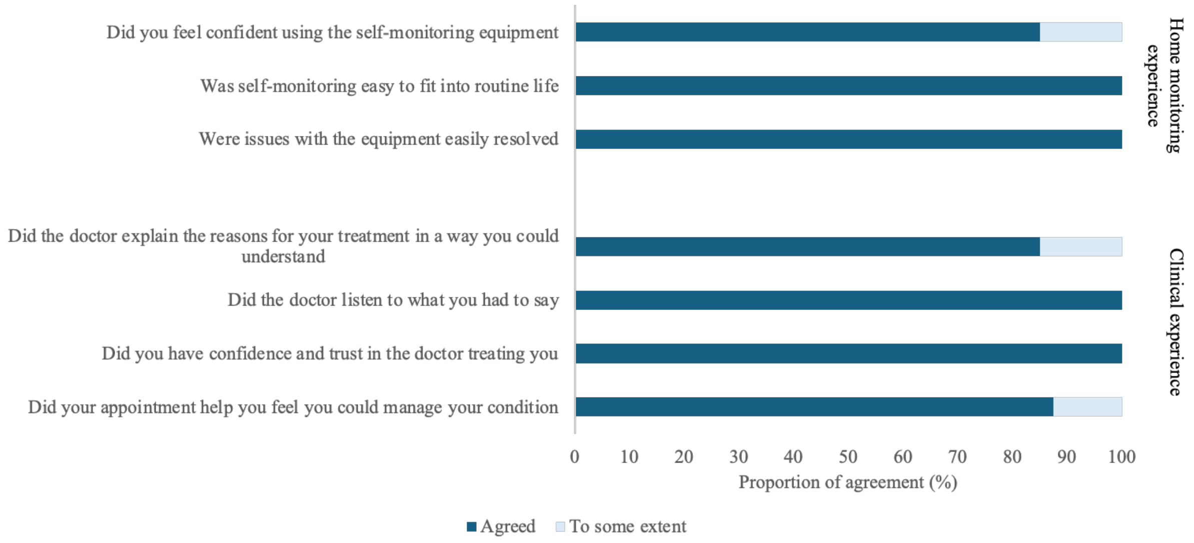


**Participants satisfaction with home monitoring and clinical care experience (% agreement with statements)**
